# The application of finite element analysis to determine the optimal UIV of growing-rod treatment in early-onset scoliosis

**DOI:** 10.3389/fbioe.2022.978554

**Published:** 2022-09-02

**Authors:** Aixing Pan, Hongtao Ding, Junjie Wang, Zhuo Zhang, Hongbo Zhang, Yuzeng Liu, Yong Hai

**Affiliations:** ^1^ Beijing Chaoyang Hospital, Capital Medical University, Beijing, China; ^2^ School of Mechanical and Power Engineering, East China University of Science and Technology, Shanghai, China

**Keywords:** early-onset scoliosis, growing-rod, proximal junctional kyphosis, finite element analysis, upper instrumented vertebra

## Abstract

**Objectives:** To analyze the stress distribution in the proximal vertebral body and soft tissue of dual growing-rod (GR) with different upper instrumented vertebra (UIV) to determine the optimal UIV.

**Methods:** A ten-year-old male EOS case treated with GR was selected. Based on spiral computed tomography (CT) scanning performed in 0.6 mm thick slices, a finite element model (FEM) of the preoperative state (M0, the original spine state) of the patient was created. Subsequently, four models with different UIV fixations were numerically analyzed by FEM, including M1 (UIV = T1, i.e., the upper-end vertebrae (UEV) of the upper thoracic curve), M2 (UIV = T2), M3 (UIV = T3) and M4 (UIV = T4, i.e., the lower end vertebrae (LEV) of the upper thoracic curve). Displacement and maximum stress in the proximal vertebral body and soft tissue were measured and compared among the five models.

**Results:** The spine model was fixed with the sacrum, and the gravity conditions were imposed on each vertebral body according to the research of Clin and Pearsall. The results are as follows:M4 model has the largest overall displacement, while M1 has the least displacement among the four models. Except M2, the maximum normalized stress of UIV increases with the downward movement of UIV. M1 has the lowerest annulus fibrosus stress and highest joint capsule stress, which is characterized by the vertebrae backward leaning, while M4 is the opposite. The supraspinous ligament stress of M3 and M4 is significantly higher than that of M1 and M2. This suggests that UIV downshift increases the tendency of the proximal vertebral bodies to bend forward, thereby increasing the tension of the posterior ligaments (PL).

**Conclusion:** The UIV of the GR is recommended to be close to the UEV of the upper thoracic curve, which can reduce the stress of the proximal PL, thereby reducing the occurrence of proximal junctional kyphosis (PJK).

## Introduction

Early-onset scoliosis (EOS) referred to as the spinal deformities in patients under 10 years of age is a complex spinal diseases difficult to solve (Hasler, 2018; Zhang and Zhang, 2020). During the period of rapid bone growth, EOS will seriously compromise the development of the spine, throacic and lungs, and even endangers life if not received optimal management in time (Senkoylu et al., 2020). There are great differences among patients with EOS in terms of the locations and types of deformation. Hence, the treatment of EOS required personalized plan due to the complexity of deformity feature.

Among the current treatment methods of EOS, the growing-rod (GR) technique, which can maximize the potential growth of the spine and deformity correction has gained great success (Cengiz et al., 2021). However, there are still complications during the process of GR treatment according to current studies, which mainly include wound infection, proximal junctional kyphosis (PJK), instrument failure, and autologous fusion (Hardesty et al., 2018; Karol, 2019). PJK refers to the progress of proximal junction angle greater than 10° after the operation, leading to severe pain, instrument-realated complication and even neurological deficits (Watanabe et al., 2016). The independent studies showed that the incidence of PJK ranged from 7% to 56% after dual growing-rods surgery (Hardesty et al., 2018).

The risk factors of PJK are mainly obtained from the retrospective study of clinical cohorts, making it difficult to directly use cadavers for experiments since the samples will gradually produce operating errors during repeated percedure (Cahill et al., 2012; Zhu et al., 2019). Although there have been many finite element models to investigate the risk factors of PJK, there is still little research on the fixed position of the growing rod, and most of which only carry out short segment modeling, without considering the stress situation in the environment of intact spine.

Thus, the C1-S1 full-spinal models of EOS patients with dual growing rod were established in the present study. The stress distribution of the proximal vertebral body and soft tissue at different levels of dual growing rods fixation was simulated by the finite element method, and the risk of PJK was analyzed, ultimately to explore the optimal upper instrumented vertebrae (UIV) of the growing rod.

## Materials and methods

### Case evaluation

**FIGURE 1:**
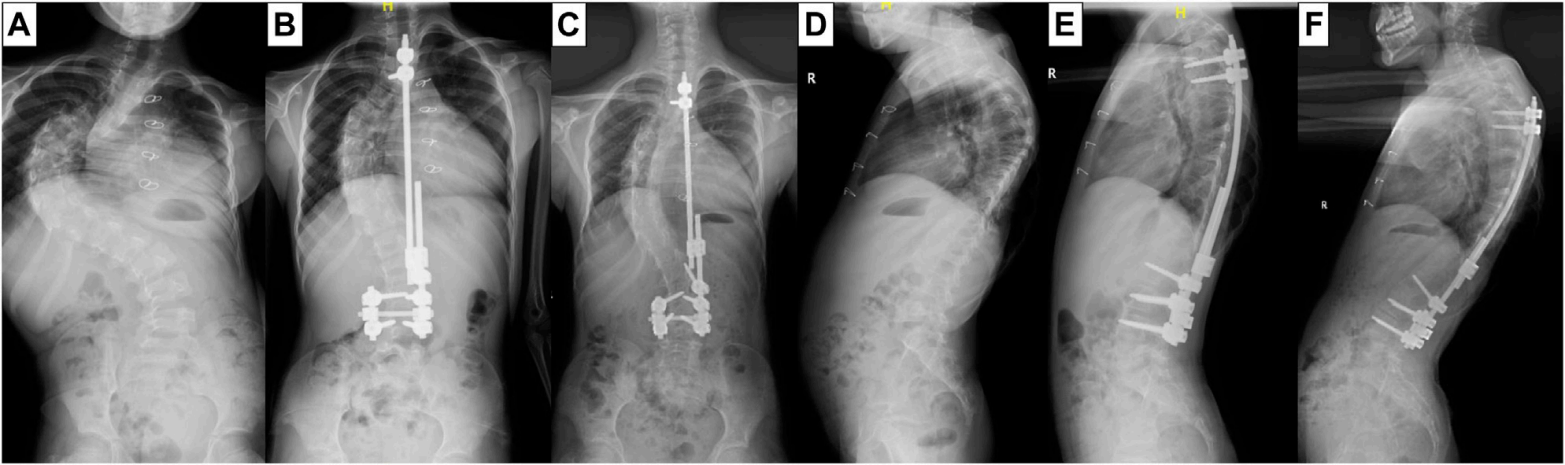
X-ray film of the patient before GR surgery **(A,D)**, after surgery **(B,E)** and at the follow-up **(C,F)**.

A 10-year-old patient with early-onset scoliosis (male, height 109 cm, weight 14 Kg) was selected. The patient underwent single growing rods surgery with T3 as the UIV and L4 as the lower instrumented vertebra (LIV) (Figure 1). The scoliosis was well corrected, but the PJK occurred in the patient during the follow-up (Figure 1F). The data were provided by Beijing Chao-yang Hospital, Capital Medical University, which included posteroanterior and lateral radiographs of patients before and after correction. The preoperative CT scan tomographic images were also provided.

### Image data acquisition

The geometry was reconstructed by 3D CT-scan images (slice thickness: 0.6 mm) from the patient, ranging from C1 to S1 segments. The CT tomographic data were stored in standard Dicom format.

### Establishment of spine 3D model

The extracted Dicom file was imported into Mimics Research 21.0 (Materialise Inc., Belgium), and the bone boundary was extracted through threshold segmentation. Each segment of the vertebra was acquired with filling and splitting and saved as a different Mask. The reconstructed vertebras were saved in STL format. The model surface was smoothed, meshed, and converted to a STEP solid model with Geomagic Studio (3D Systems Corporation, Rock Hill, South Carolina, United States). The C1-S1 segment model in STEP format was imported into Solidworks 2019 (Dassault Systèmes SolidWorks Corporation, Vélizy-Villacoublay, France) to simulate surgical correction according to the patient’s postoperative radiographs. Subsequently, cortical and cancellous bone were divided, and the intervertebral disc and facet joint capsule were established, where the intervertebral disc contained the annulus fibrosus and nucleus pulposus. The proportion of nucleus pulposus was between 30% and 50%. The thickness of cortical bone was 1 mm.

### Establishment of spine finite element model

**FIGURE 2:**
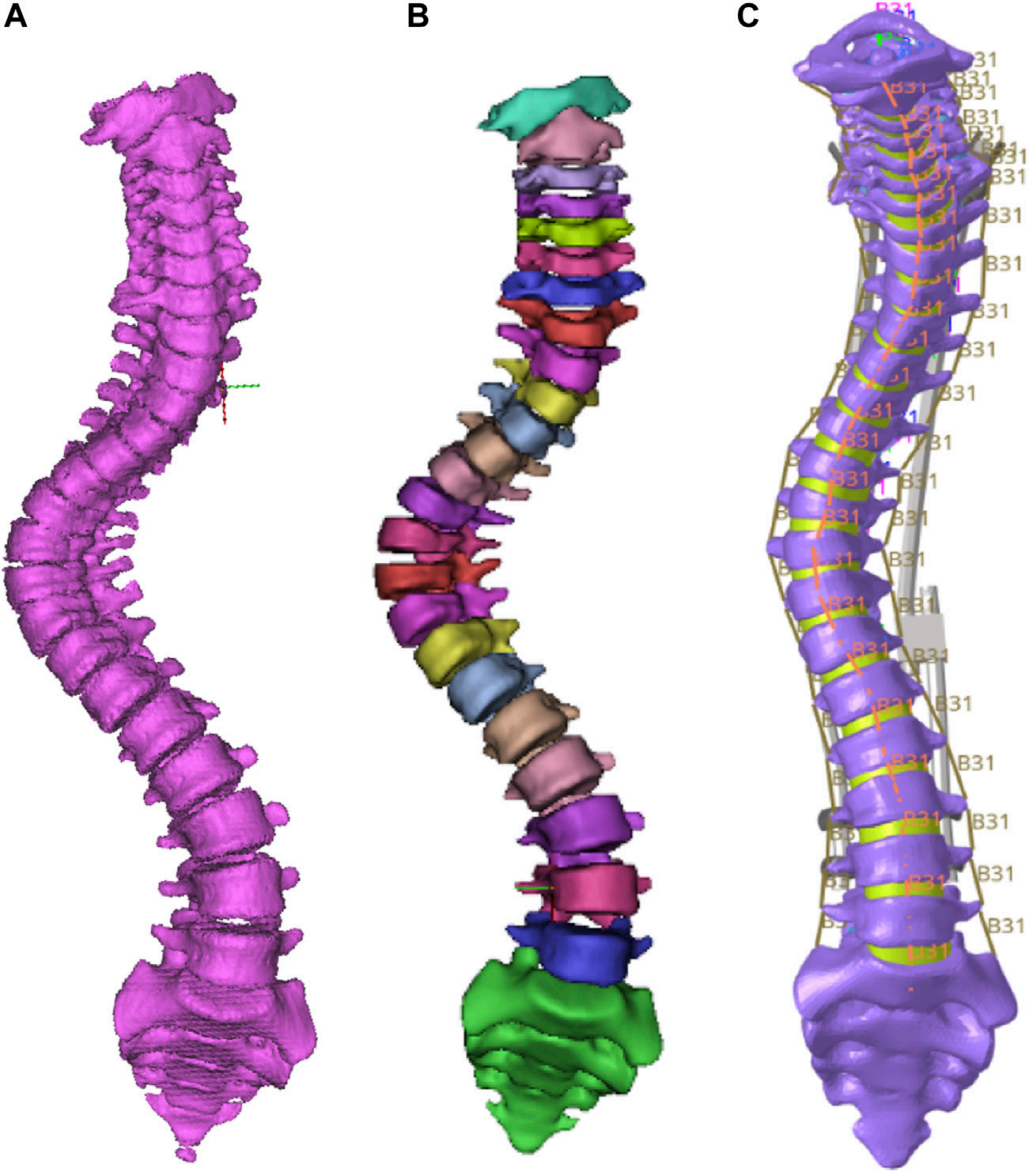
The original spine model **(A)** and FEM before **(B)** and after **(C)** meshing.

The established C1-S1 segment geometric model of the spine was imported into HyperMesh 2019 (Altair Engineering, Inc., United States) for FEM establishment. Vertebrae, intervertebral discs, and joint capsules were defined as elastic, with material properties defined in terms of elastic modulus and Poisson’s ratio (Schmidt et al., 2012; Erbulut et al., 2015; Shen et al., 2019; Kahaer et al., 2022; Liang et al., 2022). The model was meshed into tetrahedral elements (as shown in Figure 2) with 118,820 nodes and 561,469 elements. The ligaments included anterior longitudinal ligament, posterior longitudinal ligament, ligamentum flavum, interspinous ligament, supraspinous ligament, and intertransverse ligament, using a one-dimensional unit with a circular cross-section. These ligaments were subjected to tension only, and no compression (Wang et al., 2019). The material properties of each part in the spine model were shown in Table 1. Tying constraints were defined between parts of the spine. The lower part of the sacrum was fixed, and gravity was applied to the upper surface of each vertebral body to simulate the force of the spine in a standing state. Gravity value and percentage of body weight borne by the vertebral body were shown in Table 2 (Pearsall et al., 1996; Clin et al., 2011). The gravitational acceleration was 9.8 m/s^2^.

**TABLE 1:** Material properties of each part of the models.

Part	Modulus (MPa)	Poisson’s ratio *μ*	Area (mm^2^)
Cortical bone	12,000	0.30	—
Cancellous bone	500	0.20	—
Annulus fibrosus	4.20	0.30	—
Nucleus pulposus	2	0.49	—
Joint capsule	20	0.30	—
Growing rod	110,000	0.28	—
Anterior longitudinal ligament	20	0.30	38
Posterior longitudinal ligament	70	0.30	20
Ligamentum flavum	50	0.30	60
Interspinous ligament	28	0.30	35.5
Supraspinous ligament	28	0.30	35.5
intertransverse ligament	50	0.30	10

**TABLE 2:** Gravity value and percentage of body weight borne by the vertebral body.

Vertebral body	Percentage (%)	Gravity value(N)
C1	1.14	1.60
C2	1.14	1.60
C3	1.14	1.60
C4	1.14	1.60
C5	1.14	1.60
C6	1.14	1.60
C7	1.14	1.60
T1	1.10	1.54
T2	1.10	1.54
T3	5.30	7.42
T4	5.30	7.42
T5	5.30	7.42
T6	1.30	1.82
T7	1.40	1.96
T8	1.50	2.10
T9	1.60	2.24
T10	3.00	2.80
T11	2.10	2.94
T12	2.50	3.50
L1	2.40	3.36
L2	2.40	3.36
L3	2.30	3.22
L4	2.60	3.64
L5	2.60	3.64

### Analysis of different fixations

**FIGURE 3:**
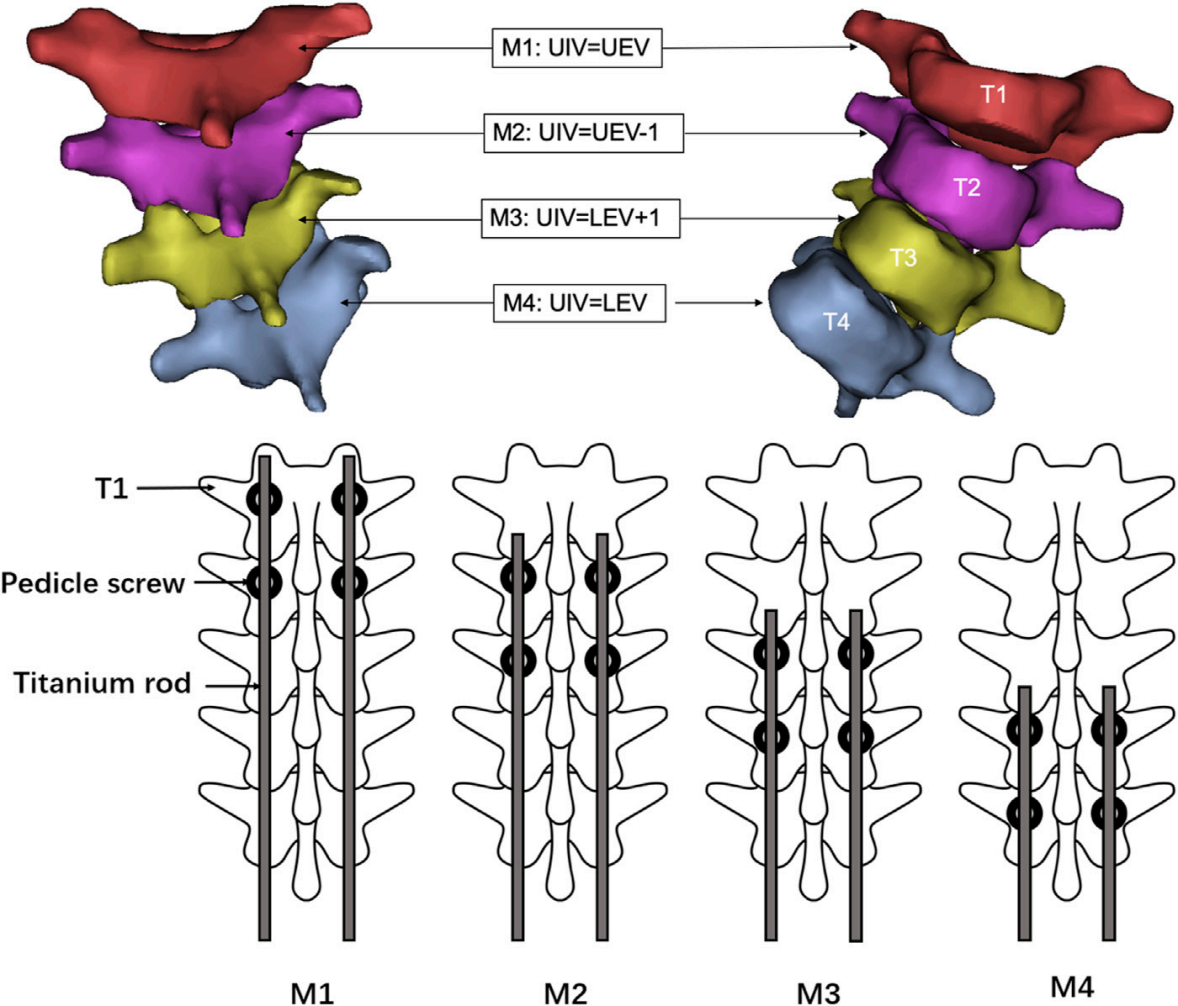
Schematic diagram of internal fixation scheme of M1–M4 model. M1:UIV = T1, i.e., the UEV of the upper thoracic curve; M2: UIV = T2, i.e., UEV-1; M3: UIV = T3, i.e., LEV+1; M4: UIV = T4, i.e., the LEV of the upper thoracic curve.

Dual growing rod fixation was adopted with four screws at the upper and lower end. The lower four screws are located at L3 and L4. Four post-orthopedic models, M1 (UIV = 1), M2 (UIV = 2), M3 (UIV = 3), M4 (UIV = 4), and one pre-orthopedic model M0 were established according to the different positions of the upper screws (Figure 3). In addition, the stress cannot be compared due to the different spatial positions of the different vertebral bodies. Therefore, according to the normalization principle, a model MC with the same spine curve after correction without using growing rods was established, to compare the stress changes before and after fusion at the same position. The finished model was solved in Abaqus.

## Results

### Model validation

**FIGURE 4:**
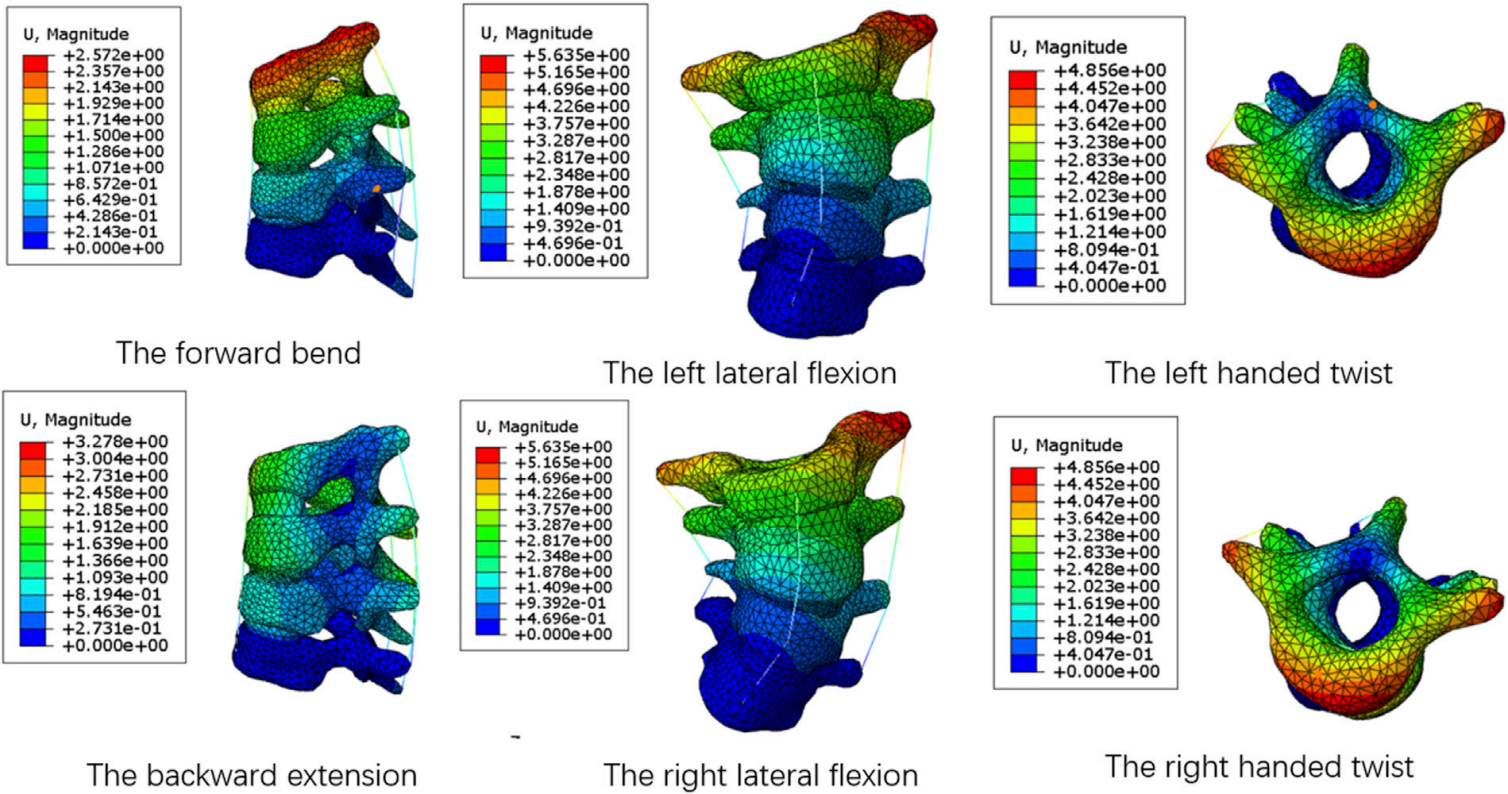
The displacement nephograms of T1–T4 segments under six motion states.

To validation the numerical simulation procedure, the average stiffness of spine segments without growth rods under the same load were compared with the literature (Busscher et al., 2009). The average stiffness refers to the ratio of the moment loaded on the spine to its angular offset, in Nm/°. The specific method was intercepting the T1-T4 segments of the complete spine model, and applying a moment of 4 N m on the upper surface of T1 to simulate six motion states of the forward bend and backward extension, the left and right lateral flexion, and the left and right handed twist. The displacement nephograms of the spine model under six motion states were shown in Figure 4. Now take the left handed twist as an example to introduce the solution of the average stiffness. The foremost point and the last point on the upper surface of the T1 were selected in Abaqus, and the coordinate values of the nodes before and after the deformation were recorded. With two straight lines before and after the deformation draw in Solidworks according to the coordinate values, the angle can be calculate. Since the offset before and after twist of the upper surface of T1 mainly occured in the parallel plane of the surface, the displacement component in the vertical plane was very small and can be ignored. Therefore, the straight lines were projected into the parallel plane of the upper surface of T1, and the angle was measured to be 7.53°.The average stiffness was the ratio of the moment value to the angle. The average stiffness values of T1–T4 segments under the six motion states were shown in Table 3.

**TABLE 3:** The average stiffness values of T1–T4 segments under the six motion states.

	Forward bend	Backward extension	Left lateral flexion	Right lateral flexion	Left handed twist	Right handed twist
Literature 1 (Busscher et al. (2009)	0.667	0.667	0.656	0.656	0.548	0.548
Our study	0.728	0.723	0.655	0.672	0.531	0.508

It can be seen from the table that the biomechanical properties of the T1–T4 segments of the spine model established in this study are not significantly different from those of the spine model in the previously recognized literature. Because the whole spine segments and the T1–T4 segments use the same modeling method and material properties, the model of the full-segment spine can be considered valid and can be used for further analysis.

### Displacement

**FIGURE 5:**
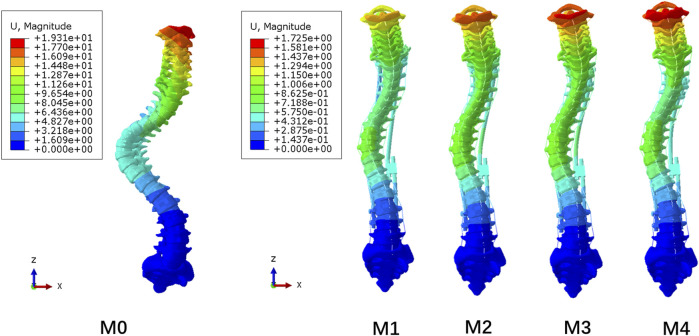
Displacement nephogram of M0–M4 model.

The overall displacements of the M0–M4 model are shown in Figure 5. The M1–M4 model was unified with the scale of the M4 model with the largest deformation. Due to the fixation of the lower surface of the sacrum, all models showed an increase in displacement from bottom to top, and the maximum displacement was located at C1. As the UIV position of the dual growing rods was shifted inferiorly, the maximum displacement of the models gradually increases, from 1.37 mm in the M1 model to 1.73 mm in the M4 model, while the M0 model without the growing rod fixation has the maximum displacement of 19.31 mm. It can be seen that the growing rod has a significant function of stabilizing the spine that can avoid a lot of displacement of the spine.

**FIGURE 6:**
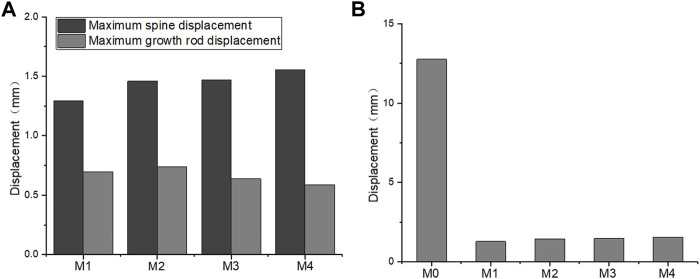
Maximum longitudinal displacement. **(A)** M1–M4 maximum longitudinal displacement of the spine and growing rods; **(B)** M0–M4 maximum longitudinal displacement of the spine.

Figure 6A shows that the longitudinal displacement of growing rods in the M1-M4 model generally decreases with the UIV shifting inferiorly. The reason may be that the length of the growing rods decreased, and the difference in the stress level was not obvious. The maximum longitudinal displacements of the five models are presented in Figure 6B, showing the same trend as the overall displacement. The fixation of longer segments can reduce the overall displacement of the spine.

**FIGURE 7:**
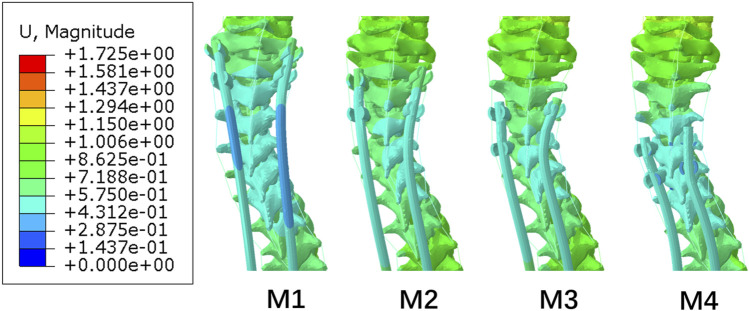
Displacement nephogram of posterior proximal of M1−M4.

Figure 7 shows the displacement of the posterior end of the M1–M4 spine. The displacement of the three to six vertebral bodies below UIV was smaller than that of other vertebral bodies. This small-displacement area changed with the UIV shifting inferiorly. This separation of displacement around UIV and UIV+1 may be related to the occurrence of PJK due to the mismatch between the stiffness of the growing rods and the spine.

### Von Mises stress

**FIGURE 8:**
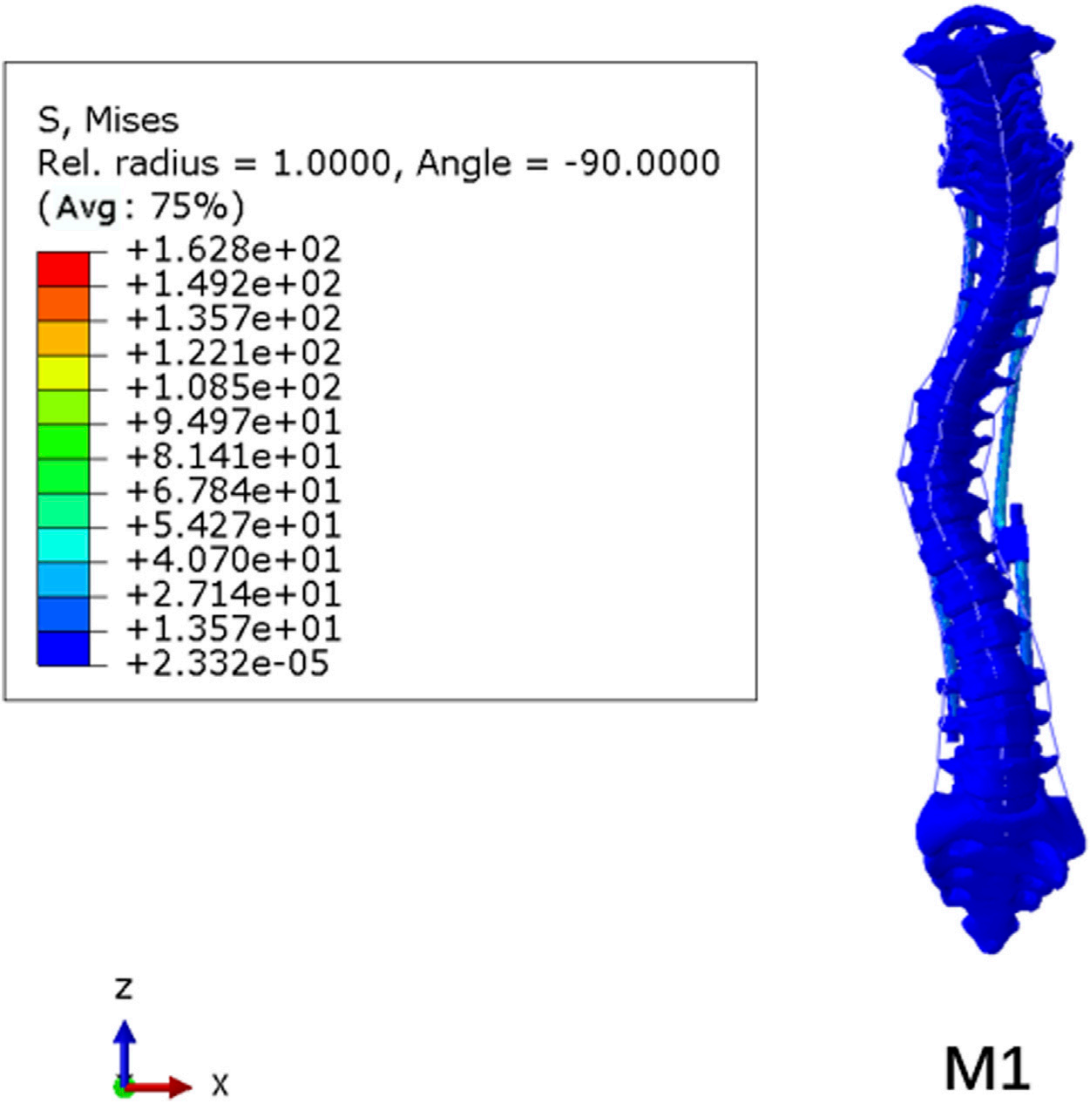
Stress nephogram of M1 model.

**FIGURE 9:**
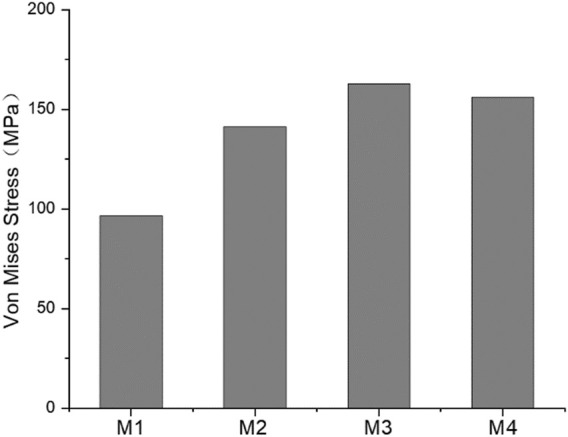
The maximum stress of M1–M4 growing rods.

The stress nephogram of the M1 model is shown in Figure 8. The stress levels in the spinal column were low, and the maximum Von Mises stress was observed on pedicle screws in all models, ranging from 96.5 to 162.8 MPa (139.15 ± 25.84 MPa). The comparison of the maximum stress of growing rods in the M1–M4 model is shown in Figure 9. The maximum Von Mises stress on UIV ranged from 10.71 to 14.01 MPa (12.36 ± 1.33 MPa), which was significantly higher than that on other vertebral bodies. The maximum Von Mises stress range on UIV+1 was 0.50–1.04 MPa (0.77 ± 0.19 MPa).

The GR fixation system had maximum stress of 162.8 MPa occurring on the pedicle screw. However, the stress was far from causing fracture to the fixation system. As the UIV position was shifted inferiorly, the maximum Von Mises stress of the pedicle screw did not show the same trend as that of UIV and UIV+1. The maximum fixation stress of the M3 model was larger than that of other models. While the maximum value of UIV stress of M2 and UIV+1 stress of M4 are the largest.

### Normalized von mises stress

The spatial position and stress concentration area of different vertebrae were completely different. UIV and UIV+1 in M1–M4 were not in the same position, which made it difficult to compare with each other. Therefore, normalization was necessary to be performed before comparison. The normalized method was to reconstruct an orthopedic spinal model (MC) without any fixation and simulate it in the light of the same pre-processing. The maximum Von Mises stress of UIV and UIV+1 of M1–M4 was divided by the maximum stress of the corresponding vertebral body of MC to obtain the normalized results of stress.

**FIGURE 10:**
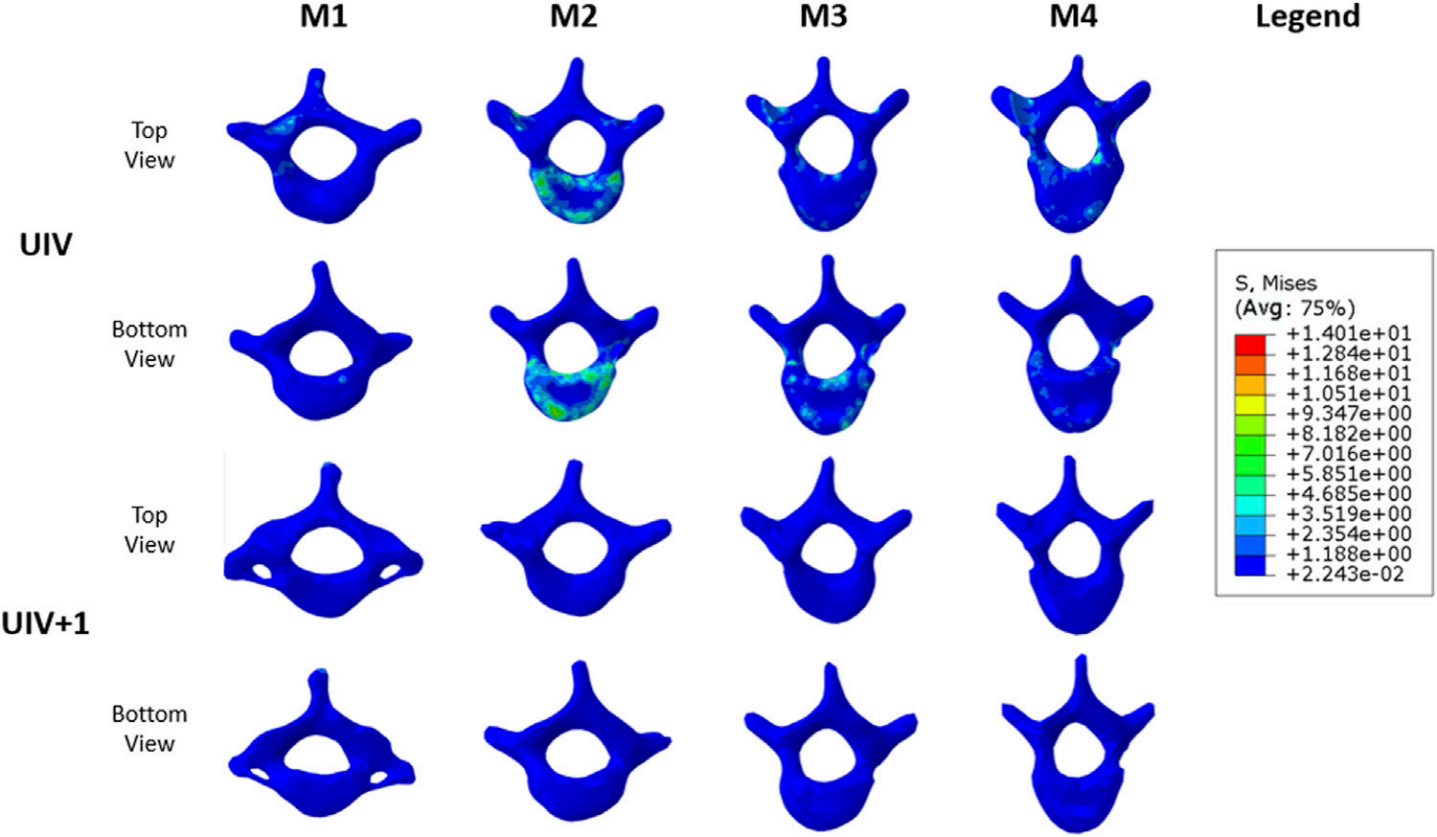
M1–M4 UIV and UIV+1 stress nephogram.

The normalized maximum value of UIV and UIV+1 stress are shown in Figure 10. Except for M1, the maximum stress of UIV increased significantly, while the maximum stress of UIV+1 decreased significantly comparing with the normalized model MC. In models with the absence of growing rods, the stress distribution of C7–T5 ranged from 4.73 to 11.05 MPa (7.88 ± 2.34 MPa), and the stress difference between UIV and UIV+1 increased significantly after fixation with growing rods. The stress differencebetween UIV and UIV+1 in M2 (T1, T2) was the largest, followed by M4 (T3, T4), and the smallest is M1 (C7, T1). Even before normalization, the Von Mises stress of UIV in M2 was the largest among the four groups.

**FIGURE 11:**
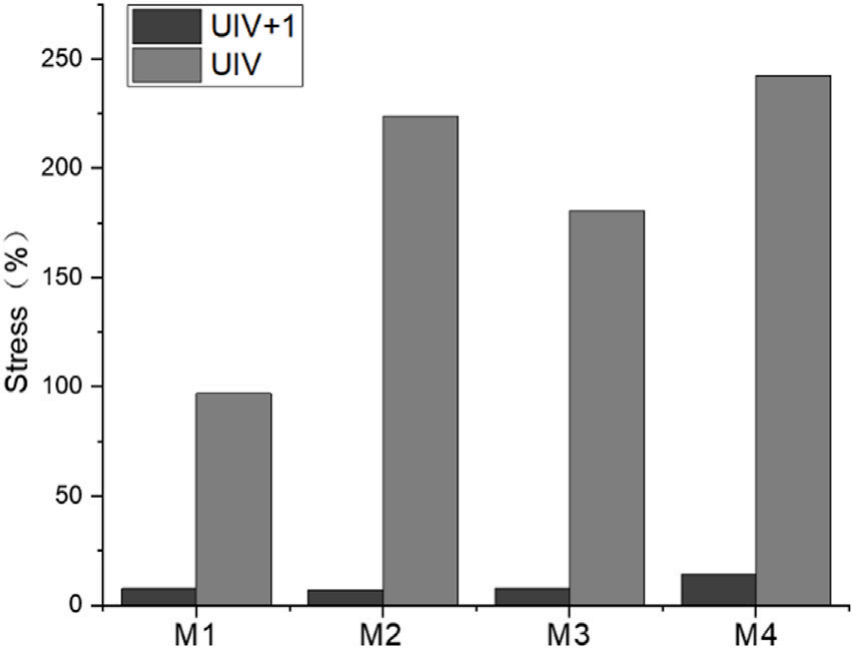
UIV and UIV+1 stress nephogram of M1–M4 model.

According to the stress changes of the vertebral body, the dual growing rods and pedicle screw fixation system can increase the stress of UIV and reduce the stress of UIV+1. The stress distribution of the vertebral body is shown in Figure 11. The stress distribution maps of UIV and UIV+1 were compared under the same scale. The stress of UIV+1 was all below 1.19 MPa, and the stress in most areas of UIV was low, while a few stress concentrated areas were mainly in the contact part of the vertebral body and annulus fibrosus, joint capsule connection, and the connection part of vertebral body and screw. There were large areas of stress concentration in both the upper and lower vertebral bodies of the UIV in M2, and the stress level was significantly higher than that of the other three groups (Figure 11). The stress in the M1 model was significantly lower overall, with only a small stress concentration at the pedicle and pedicle screw fixation sites. The stress on the lower surface of the UIV vertebral body in M3 was more concentrated than that on the upper surface, while that on M4 was the opposite.

The stress distribution results were better than other groups when UIV was T1. M1 was a better solution because not only was the maximum stress of UIV almost unchanged before and after fixation but there was no stress concentration on the upper and lower surface of the vertebral body. Excluding M2, the maximum normalized stress value of UIV increased with the downward movement of UIV, while the value of M2 was close to that of M4, which may be related to the spinal curve of the case.

### Annulus fibrosus and joint capsule

**FIGURE 12:**
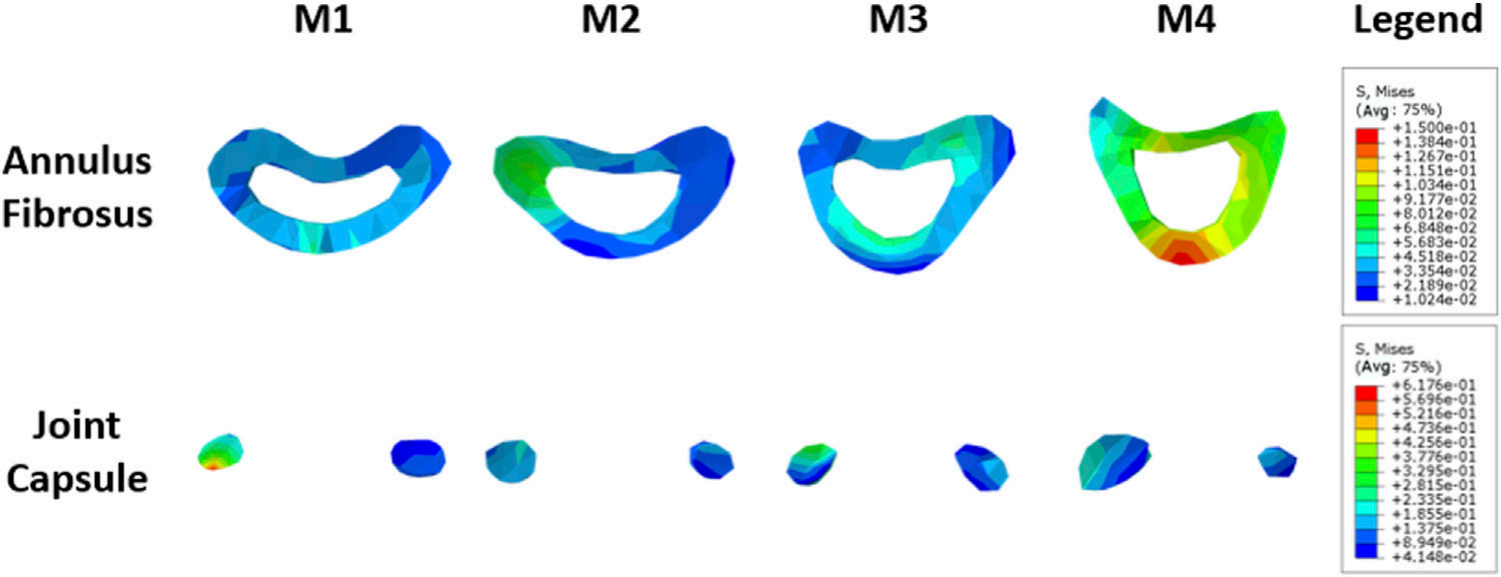
Stress nephogram of annulus fibrosus and joint capsule.

**FIGURE 13:**
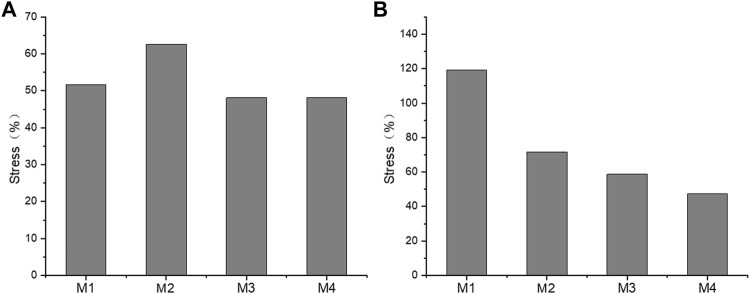
The maximum stress of fiber ring **(A)** and joint capsule **(B)** of the M1–M4 model.

The annulus fibrosus and joint capsule stresses associated with UIV and UIV+1 were also compared. Figure 12 shows the stress distribution of the annulus fibrosus and joint capsule proximal to the device. Figure 13 exhibits the comparasion results of the normalized Von Mises stress maxima of the annulus fibrosus and joint capsule. The fixation with growing rods reduced the maximum stress in M1, M3, and M4 by about half, and in M2 by about 40%. The Von Mises stress of M2–M4 decreased to 72%, 59%, and 48%, respectively, while the Von Mises stress of M1 increased to 119%. The joint capsule stress of M1–M4 showed a downward trend after normalization. The comparison of the fiber ring stress cloud showed that the stress level of M2 and M3 was higher than that of M1, while the stress of the four models was almost greater than that of M1 overall, and the maximum stress appeared in the front end. The stress peak of the joint capsule appeared at UIV = T1, but there was no significant difference among the other groups.

### Interspinous ligament and supraspinous ligament

**FIGURE 14:**
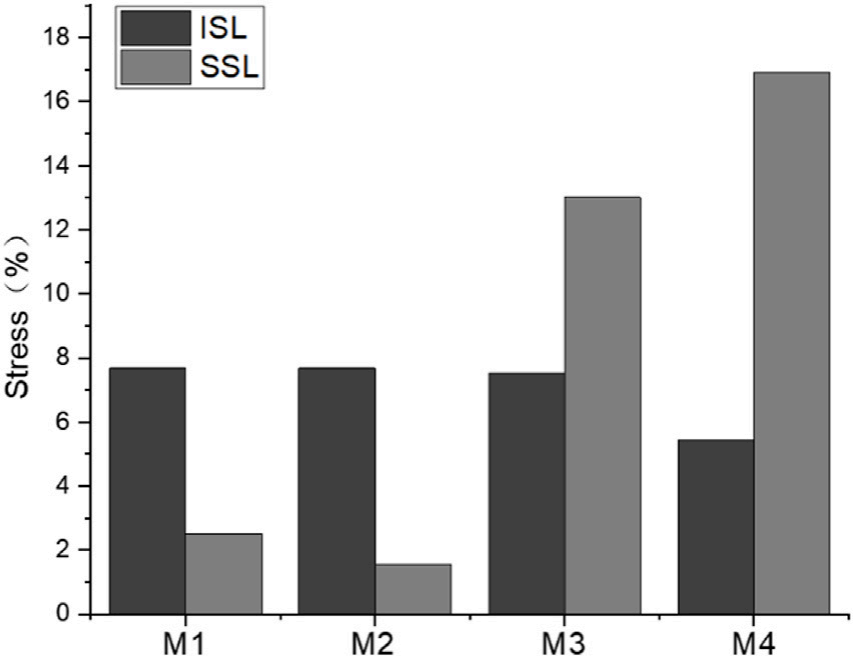
Maximum ISL and SSL stress of M1–M4 model.

The normalized stress of the interspinous ligament (ISL) and supraspinous ligament (SSL) is shown in Figure 14 **.** The stress of ISL after normalization was significantly lower than that of SSL before fixation with growing rods, with ISL stress of 5.4%–7.7% and SSL stress of 4.6%–16.9%. The difference between groups was significant. The ISL stress variation of M1–M3 was similar, while the stress of M4 was lower. The SSL of M1 and M2 were almost free from stress with the influence of growing rods, but the SSL stress of M4 was the largest.

## Discussion

EOS is a spinal deformity with the characteristic of early occurance, rapid progression, and continuous growth of the spine, with a significant risk of cardiopulmonary insufficiency (Chang et al., 2021; Zhang et al., 2022). Patients with severe spinal curvature usually require spinal correction with growing rods after the failed attempts at conservative treatment (Akbarnia et al., 2022; Heffernan et al., 2022). Compared with single growing rods, dual growing rods system contributes to a more stable constrution for spine (Urbanski et al., 2020; Cengiz et al., 2021). It can provide a stronger control force and reduce the mechanical stress of the instrumentation. However, dual growing rods and pedicle screw fixation, the most stable and effective option, may be more likely to lead to PJK (Pan et al., 2018; Ogura et al., 2021). Clinical studies have shown that there are many factors associated with PJK, but the relationship between the position of pedicle screw fixation and PJK is still a controversial topic (Homans et al., 2020). PJK occurs at the proximal end of orthopedic instruments and is closely related to local stress concentration after surgery (Erkilinc et al., 2022). Therefore, the stress quantity of the functional spine unit (FSU) composed of UIV and UIV+1 is mainly concerned, including the stress of UIV and UIV+1, annular fiber, joint capsule, interspinous and supraspinal ligaments. In addition, the morphological variables of the spine were compared.

Finite element models have been introduced to investigate the biomechanical behavior of the spine and provide information cannot be easily obtained through *in vivo* and *in vitro* experimental studies, such as stress distribution and displacement under static conditions (Rohlmann et al., 2006; Zhu et al., 2019). Despite these successful outcomes, that segmental spinal model has natural shortcomings. An intact spine model used in finite element analysis could provide a more comprehensive and close-to-real simalution in the dynamical investigation of the biomechanical behavior of the spine. The biomechanical modeling and simulations of an intact scoliotic spine can effectively help surgeons assess and evaluate the appropriateness of various instrumentation scenarios, and accordingly find an optimal solution to maximally correct the scoliotic spine and avoid complications (Robitaille et al., 2009; Majdouline et al., 2012; Jalalian et al., 2013). Thus, an intact spinal was applied in the finite element modeling to determine an optimal UIV of growing-rod to minimalize the risk of PJK in the present study.

The surgically induced changes in the UIV are an important parameter associated with the development of PJK (Homans et al., 2020). Our study shows that as the UIV position of the dual growing rods is shifted inferiorly, the maximum displacement of the models gradually increases, therefore the fixation of longer segments reduces the overall displacement of the spine. Simultaneously comparing the UIV and UIV+1 stress nephogram of the four models, it is found that the dual growing rods and pedicle screw fixation system can increase the stress of UIV and reduce the stress of UIV+1. By analyzing the UIV stress nephogram, it seems that the stress concentration areas of the vertebral body are mainly in the contact part of the vertebral body and annulus fibrosus, joint capsule connection, and the connection part of the vertebral body and screw. The stress results of the vertebral body show that the stress in the M1 model is significantly lower overall, with only a small stress concentration at the pedicle and pedicle screw fixation sites. Therefore the stress distribution results are better than other groups when UIV is T1, which is the UEV of the upper thoracic curve.

Intervertebral disc injury and degeneration is a risk factor for PJK (Yagi et al., 2011), and the pressure on the annulus fibrosus may lead to annulus fibrosus injury or failure (Iatridis and ap Gwynn, 2004). Combined with the stress distribution of the annulus fibrosus and the joint capsule, it can be seen that M1 has lower annulus fibrosus stress and higher joint capsule stress, while M4 has the opposite result. The tendency of the former was vertebrae backward leaning, while the latter was vertebrae forward-leaning. The deformation trend of UIV and UIV+1 of the M4 model is consistent with the development of PJK (Pan et al., 2018). The result suggests that UIV downshift may be more likely to lead to PJK.

Posterior Ligament Complex (PLC) consists of ISL, SSL, and facet capsular ligaments, which play an important role in maintaining spinal stability and limiting the normal range of movement of the spine (Radcliff et al., 2012). Some researchers believe that the ligamentum flavum should be part of PLC, the damage of which during surgery is also recognized as a risk factor for PJK (Hostin et al., 2013). Therefore, the stress levels of SSL and ISL are of great significance to analyze the stress of the spine. The results showed that the growing rods significantly reduced the stress of each ligament, but this may stem from that the model used in the study was not a normal healthy spine, and the stress of the PL in the MC model was already higher than normal. However, the comparison between each group of models shows that the SSL stress of M3 and M4 is significantly higher than that of M1 and M2. This indicated that the deformation trend of UIV and UIV+1 vertebrae was bending forward in M3 and M4, which was consistent with the analysis results of the annulus fibrosus and joint capsule.

Our study has several limitations. First, the EOS spine FE model was developed based on the geometric information of the spine from a single EOS patient, which cannot calculate the statistical significance. Then, the paraspinal muscles were not constructed in this model, although a widely recognized physiological follower load was applied to simulate the effect of muscle force (Yu et al., 2016; Wong et al., 2020). Nevertheless, the follower load could not entirely replace the muscle functions, which might have more complex contributions to spinal stability. Besides, the FE models were constructed without considering the degenerative and deformity changes such as facet hyperplasia, annular tearing, endplate sclerosis, or vertebral osteoporosis, which may make the conclusion less persuasive.

## Conclusion

In the current study, the stress distribution in the proximal vertebral body and soft tissues of dual growing rods with different proximal fixation segments were analyzed using FEM to explore the optimal UIV. The results showed that the stress of the vertebra and soft tissue at M1 was the lowerest, and the fixed condition could conquer the PJK formation and progression. M4 model, on the contrary, is more likely to cause PJK occurrence. Therefore, PJK is less likely to occur when the upper end of the dual growing rods is fixed close to the UEV of the upper thoracic curve.

## Data Availability

The raw data supporting the conclusion of this article will be made available by the authors, without undue reservation.
